# The Diagnostic Accuracy of Optical Coherence Tomography Angiography for Neovascular Age-Related Macular Degeneration: A Comparison with Fundus Fluorescein Angiography

**DOI:** 10.1155/2016/7521478

**Published:** 2016-03-23

**Authors:** Jingwen Gong, Suqin Yu, Yuanyuan Gong, Fenghua Wang, Xiaodong Sun

**Affiliations:** ^1^Department of Ophthalmology, Shanghai General Hospital of Nanjing Medical University, Shanghai 200080, China; ^2^Department of Ophthalmology, Zhejiang Provincial People's Hospital, 158 Shangtang Road, Hangzhou, Zhejiang 310014, China; ^3^Department of Ophthalmology, Shanghai First People's Hospital, School of Medicine, Shanghai Jiao Tong University, Shanghai 200080, China

## Abstract

*Purpose.* To describe the morphological characteristics and efficacy of OCTA in detecting CNV in nAMD. We retrospectively reviewed 53 patients (86 eyes) with suspected CNV secondary to wet AMD. All the patients underwent a multimodal assessment for CNV. Two independent readers calculated the sensitivity and specificity of OCTA in detecting CNV compared with FA. A qualitative analysis of OCTA was also performed to describe the morphological appearance of CNV. Among 86 eyes of 53 patients, 52 eyes were diagnosed as having CNV based on the FA imaging analysis. According to FA, CNV was classified as classic in 28 eyes, predominantly classic in 6 eyes, minimally classic in 9 eyes, and occult in 9 eyes. In 56 eyes, CNV was visualized on OCTA and corresponding OCT B-scans. In total, 46.4% (26/56) had well-circumscribed vessels, and 53.6% (30/56) showed poorly circumscribed vessels. There were 11 false positives and 7 false negatives using OCTA. The specificity of OCTA for the detection of CNV was 67.6%, with sensitivity of 86.5%. OCTA may help in the noninvasive diagnosis of CNV and may provide a method for monitoring the evolution of CNV.

## 1. Introduction

Neovascular age-related macular degeneration (nAMD), an advanced form of macular degeneration, is the leading cause of visual impairment in older adults related to AMD [1]. The presence of abnormal blood vessels, known as choroidal neovascularization (CNV), can penetrate Bruch's membrane (BM) and extend into the subretinal pigment epithelial (RPE) or subretinal space. CNV can induce hemorrhage, fluid exudation, and fibrosis, resulting in photoreceptor damage and vision loss [2]. To prevent progressive, irreversible vision loss, early visualization and diagnosis of the CNV lesion are essential.

As the current gold standard of determining the presence of leakage [3], fluorescein angiography (FA) can provide dynamic information (transit time of dye to travel to the eye). Leakage of dye in the later frames of the angiogram is used to diagnose and classify CNV as classic, occult, or combination subtype. However, it is an invasive procedure, requiring intravenous dye injection, which can induce discomfort, nausea, and occasionally anaphylaxis [4, 5]. In addition, this technique is time consuming, taking about 15–20 min to complete, which can limit its routine use in a busy clinical setting.

For these reasons, optical coherence tomography (OCT) was introduced and has become a widely used noninvasive imaging technique to detect the presence and activity of CNV without the use of intravenous dye. It enables visualization of the morphological features of the fibrovascular complex and the exudative consequences of fluid accumulation, which is accompanied by retinal thickening and edema [6]. However, OCT is sensitive only to the backscattering light intensity and cannot distinguish vasculature from fibrous and other surrounding tissues. Due to this limitation, the precise location and activity of the CNV cannot be determined. Thus, OCT imaging cannot replace but supplement FA in the diagnosis of nAMD [7].

Optical coherence tomography angiography (OCTA) is a novel imaging modality that allows direct visualization of the retinal and choroidal vasculature in vivo. In OCTA, high-frequency and dense volumetric scanning are applied to detect blood flow by analyzing the signal decorrelation between scans. Compared with stationary areas of the retina, the movement of erythrocytes within a vessel generates a decorrelated signal [8]. Unlike traditional angiography, OCTA does not require the use of exogenous dyes, thus avoiding potential side effects, such as nausea or other more serious adverse events.

However, the role of OCTA as a diagnostic tool has not been widely investigated. Specifically, very few clinical studies [9] have evaluated the accuracy of OCTA imaging for the diagnosis of nAMD. Therefore, we designed this study to evaluate the efficacy of OCTA in detecting nAMD compared to the current gold standard, FA.

## 2. Materials and Methods

### 2.1. Study Population

This observational case study adhered to the tenets of the Declaration of Helsinki and was approved by the Eye Research Institute of Shanghai Jiao Tong University, Shanghai, China. We retrospectively reviewed 53 consecutive patients (86 eyes) with maculopathy who visited the clinic at the Department of Ophthalmology, Affiliated First People's Hospital of Shanghai Jiao Tong University, between January 2015 and July 2015. All the patients underwent a comprehensive eye examination, which included slit-lamp biomicroscopy, color fundus photography, FA, spectral-domain OCT (SD-OCT), and OCT angiography. The subjects included in this study were (1) patients over 50 years with clinical features of age-related maculopathy, such as soft or hard drusen and pigmentary alterations and (2) macular exudative signs on at least one of the two imaging examinations (FA or SD-OCT). The exclusion criteria included (1) patients without OCTA or FA results available for analysis or the OCTA/FA not being performed within 7 days of each other; (2) patients with CNV secondary to pathological myopia, angioid streaks, chorioretinitis, central serous chorioretinopathy, tumors, or trauma; and (3) media opacities, such as cataracts, preventing detailed imaging. To make our results comparable to real-world clinical practice, patients who were treated with intravitreal antivascular endothelial growth factor therapy (VEGF) and thought to have new CNV lesions were included in our study. A small number of individuals may have had mild or moderate myopia (<−6.0 D), and these were not excluded from the analysis.

### 2.2. Image Processing

The SD-OCT and FA images were acquired using the Spectralis HRA + OCT (Heidelberg Engineering, Heidelberg, Germany). The software has an automatic real-time (ART) function to reduce noise and improve the image quality. With ART activated, multiple frames (B-scans) of the same scanning location are taken. The SD-OCT protocol included multiple horizontal and vertical tomographic linear and volume scans centered on the macula in the raster mode and/or in the center of the suspected lesion. According to a standardized acquisition procedure, the FA images were assessed as follows: Infrared Radiation (IR) imaging and FA documentation of initial (0–45 sec), middle (1-2 min), and late (more than 10 min) phases of the macular area after the injection of 2.5 mL of fluorescein.

The OCT angiography images were acquired using the prototype AngioVue OCTA system on the commercially available Avanti SD-OCT device (Optovue Inc., Fremont, CA). This system uses a split-spectrum amplitude-decorrelation angiography (SSADA) software algorithm to obtain images in approximately 2.6 sec and has an A-scan rate of 70,000 scans per second, and it uses a light source centered on 840 nm and a bandwidth of 45 nm [10]. Orthogonal registration and the merging of two consecutive image sets were used to perform motion correction to minimize motion artifacts arising from microsaccades and fixation changes [11]. Three-dimensional OCT angiograms were coregistered with the cross-sectional OCT B-scans, allowing visualization of both the retinal flow and structure in tandem.

We obtained 3 × 3-mm and 6 × 6-mm OCT angiograms centered on the macula to ensure that more peripheral CNV lesions were not missed. The OCTA software was used to manually alter the automated segmentation and its relative depth in the retina and choroid. Two automated segmentation lines referencing the outer retina on the coregistered OCT B-scans were manually fine-tuned to be located at the outer aspect of the inner nuclear layer (inner boundary) and the level of Bruch's membrane (outer boundary) [12]. The inner boundary was adjusted to include the innermost region with suspected CNV (characterized by interruptions in the RPE, the presence of a subretinal and/or sub-RPE hemorrhage, lipids, RPE detachment, subretinal or intraretinal fluid, or subretinal hyperreflective material). The outer boundary was adjusted to lie directly anterior to Bruch's membrane, so that the minimal choroidal vasculature was included in the region imaged by OCTA [9].

### 2.3. Image Analysis

Two independent and trained readers evaluated each set of images (IR, FA, SD-OCT, and OCTA). The readers were blinded to any clinical patient information, such as the patient's history, visual acuity, and which eye was the index eye, if not both. If there was disagreement between the two readers, a third ophthalmologist was asked to adjudicate.

For FA, according to the criteria of the Macular Photocoagulation Study [13, 14], the CNV lesions were graded as classic, occult, and combination, which included the predominantly classic and minimally classic subtypes. Classic CNV was defined as an area of uniform and early (<30 sec) hyperfluorescence leakage throughout the middle and late phases. Occult CNV was identified by fibrovascular pigment epithelial detachment (stippled hyperfluorescence) or late-phase leakage of an undetermined source.

The appearance of CNV on the OCTA images and coregistered corresponding OCT B-scans was assessed, in addition to the presence of subretinal fluid, intraretinal fluid, or sub-RPE fluid. CNV was defined as the presence of a decorrelation signal at the outer-retina level on OCTA consistent with the vascular component of the lesion. The appearance of CNV on an OCTA image was classified as either well-circumscribed (lacy wheel or sea fan-shaped vessels) or poorly circumscribed (long filamentous vessels).

### 2.4. Statistical Analysis

Statistical analysis was performed with PASW Statistics for Windows, Version 18.0 (SPSS, Inc., Chicago, IL).

## 3. Results

In this study, we reviewed 53 patients (106 eyes) with macular degeneration who visited the clinic at the Department of Ophthalmology. Twelve eyes were excluded because of poor-quality images attributable to poor fixation or media opacity. Eight eyes were excluded because of the absence of OCTA or FA results. Twenty-eight eyes were treated with intravitreal anti-VEGF therapy for CNV, and these were not excluded from the analysis. Within this group, 86 eyes of 53 patients were assessed. The patients consisted of 33 men and 20 women aged between 50 and 85 years (mean, 67 years). Fifty-two eyes were diagnosed as having CNV with FA, with five patients diagnosed as having bilateral nAMD. According to FA, the CNV lesions were classified as classic in 28 eyes, predominantly classic in 6 eyes, minimally classic in 9 eyes, and occult in 9 eyes.

**Figure 1:**
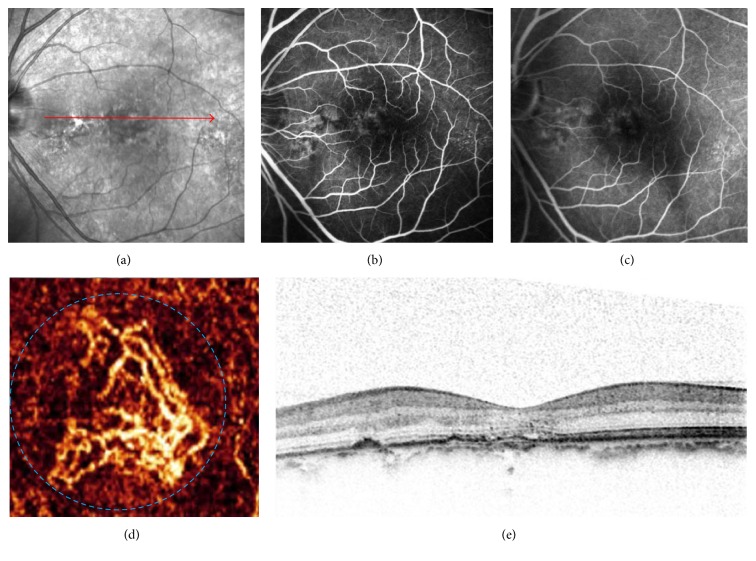
Images of choroidal neovascularization (CNV) observed in false-positive case on optical coherence tomography angiography (OCTA). (a) IR imaging from a 55-year-old man who was previously treated with 3 intravitreal anti-VEGF injections. (b and c) Early- and late-frame FA images of the patient displaying mild hyperfluorescence that is stable throughout the FA in the region of CNV without pooling. (d) A 3*∗*3-mm En face angiogram of the outer retina showing a well-circumscribed CNV in the subfoveal (blue dashed circle). (e) Intraretinal cystic spaces and mild RPE irregularity seen on the spectral-domain optical coherence tomography occur at matching locations on IR imaging (red line).

**Figure 2:**
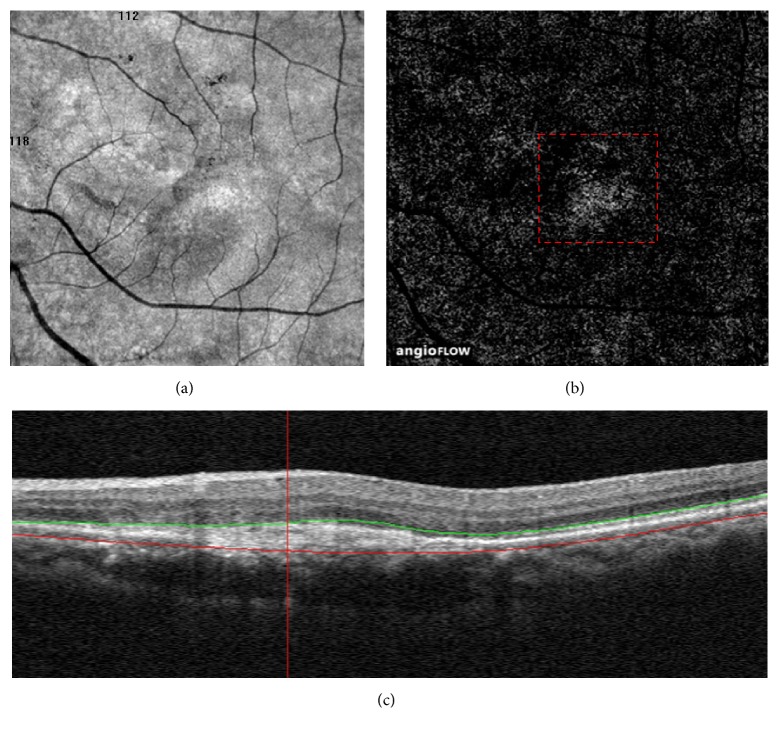
Distinguishing the choriocapillaris artifact from choroidal neovascularization (CNV). (a and b) A 6*∗*6-mm En face angiogram of the outer retina from a 62-year-old woman who were previously treated with 18 intravitreal anti-VEGF injections showing a homogenous and ill-defined network of vessels with a granular appearance (red dashed box). (c) Spectral-domain optical coherence tomography displaying a fusiform or spindle-shaped complex of high reflectivity located within the subretinal space.

**Figure 3:**
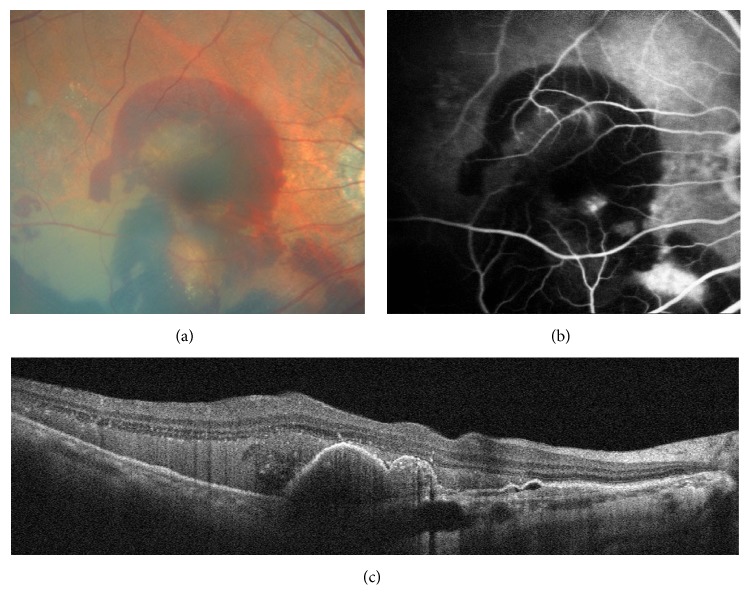
Images of subretinal hemorrhage observed in false-negative case on optical coherence tomography angiography (OCTA). (a) Color fundus photograph of the patient showing large amounts of subretinal hemorrhage. (b) Late-frame FA image displaying big leakage and pooling at the inferonasal to the macula together with posterior pole pigment epithelium detachment, also with masking effect attributable to subretinal hemorrhage. (c) Spectral-domain optical coherence tomography (SD-OCT) demonstrating large amounts of subretinal hemorrhage over the retinal pigment epithelial (RPE) detachment.

The qualitative tomographic OCTA review showed signs of CNV in 56 eyes. Eleven false-positive cases were observed on OCTA (Table 1). In six eyes that were previously treated with three or more intravitreal anti-VEGF injections, the patients had smaller but still notable CNV in the outer-retina segmentation of the OCTA but no apparent CNV on FA or detectable fluid accumulation on coregistered OCT B-scans (Figure 1). Chronic disciform scars (i.e., inactive CNV scars) were detected in 5 of 11 OCTA images with FA, but these appeared on OCTA as a homogenous and ill-defined network of vessels, with a granular appearance at the outer-retina segmentation (Figure 2). There were seven false-negative cases, and three of seven OCTA images showed a large amount of subretinal hemorrhage (Figure 3). The remaining images were of poor quality due to the motion artifacts and were believed to have no identifiable features of active CNV on OCTA. The specificity of OCTA for the detection of CNV was 67.6%, with a sensitivity of 86.5% and positive and negative predictive values of 80.4% and 76.7%, respectively (Table 2).

**Figure 4:**
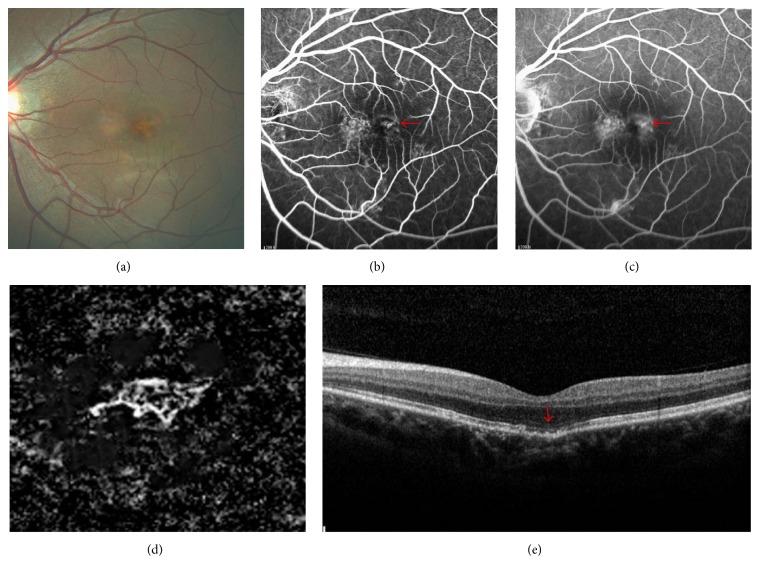
Multimodal imaging of neovascularization in age-related macular degeneration. (a) A color fundus photograph image from a 50-year-old male patient who complained of the reduced vision for one week displaying the presence of drusen in the subfoveal. (b) Early-frame FA showing hyperfluorescence in the region of CNV (red arrow). (c) Late-frame FA image showing leakage and pooling (red arrow). (d) A 3*∗*3-mm En face angiogram of the outer retina demonstrating a small well-circumscribed CNV in the subfoveal surrounded by a halo of choriocapillaris vessels. (e) Spectral-domain optical coherence tomography (SD-OCT) only showing drusen, disrupted of retinal pigment epithelium (RPE), and small defect of the photoreceptor layer which were graded as dry AMD (red arrow).

**Figure 5:**
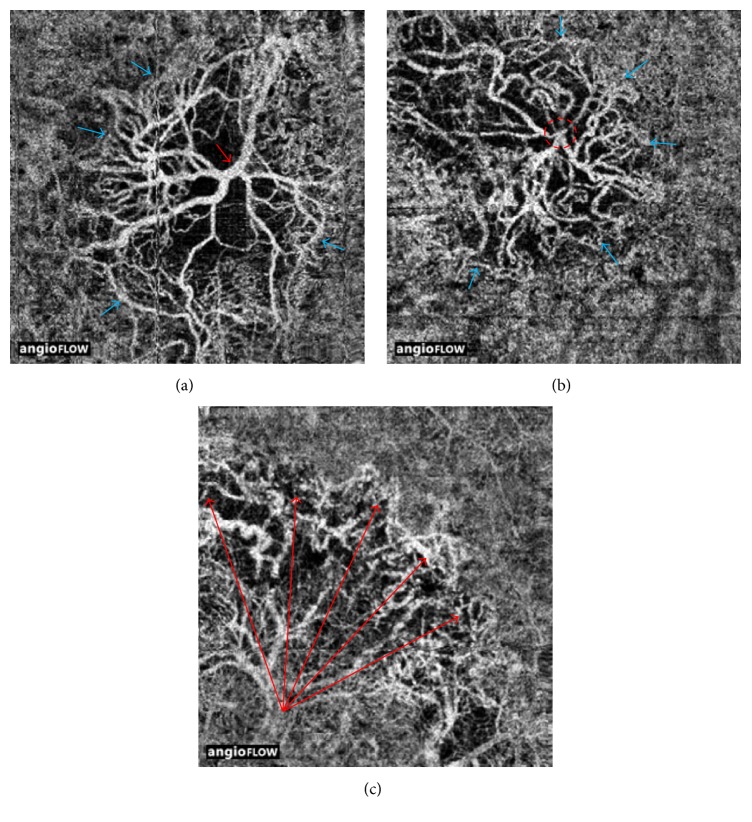
Optical coherence tomography angiography (OCTA) of treated choroidal neovascularization. (a) A 3*∗*3-mm OCTA en face projection image from an 84-year-old man who was treated with 20 intravitreal anti-VEGF injections. Note the large diameter of the trunk vessels (red arrow). At the periphery there appears to be a defined anastomotic connection around the border (blue arrows). (b) A 3*∗*3-mm En face angiogram of the neovascular membrane from a 65-year-old man who were previously treated with 18 intravitreal anti-VEGF injections showing the red dashed circle that encompasses the trunk vessels of the lesion. Note the neovascular complex with vessels radiating in all directions from the center and the terminal loops of the vessels (blue arrows). (c) A 3*∗*3-mm OCTA en face projection image from a 65-year-old man who were treated with 16 intravitreal anti-VEGF injections. Note the long extent of the vessels as they reach the edge of the lesion (red arrows).

In the 56 eyes with CNV associated with nAMD, 26 eyes had well-circumscribed vessels in the CNV area, and 30 eyes showed poorly circumscribed vessels on the OCTA images. We found four eyes at the transition point between dry and nAMD. Among these, one patient complained of reduced vision in both eyes for one week. Drusen deposits, disrupted of RPE, and defect of the photoreceptor layer were visible on OCT B-scans. A decorrelation signal was noted at the outer-retina level on OCTA. The FA also showed hyperfluorescence in the region of the CNV (Figure 4). The images of another case showed mild drusenoid pigment epithelial detachment (right eye) and a macular membrane with eccentric lesions (left eye) without detectable fluid accumulation on the OCT B-scans, but we found the presence of a decorrelation signal at the outer-retina level on OCTA and a leaky CNV in the late phase of FA. In 12 eyes that were previously treated with more than 10 intravitreal anti-VEGF injections, the vascular networks shared similar characteristics, with prominent vascular loops and anastomotic connections (Figure 5). Eight of the 12 eyes had large main trunk vessels, with the vessels radiating in a branching pattern, in all directions either from the center of the lesion or from one side of the lesion.

## 4. Discussion

FA can detect dynamic patterns of dye transit and leakage and keeps the current gold standard for diagnosing CNV [3]. However, traditional angiography is invasive and time consuming. Other major limitations are that it provides only a two-dimensional image and cannot directly visualize nascent vessels. SD-OCT is increasingly used in clinical practice to determine both the presence and activity of CNV. However, it cannot replace FA as the gold standard in the diagnosis of nAMD, because the reflectivity of CNV tissue and drusenoid material, hemorrhages, and RPE are similar on OCT. Therefore, it is highly desirable to develop a novel method, such as OCTA, for monitoring nAMD. OCTA can simultaneously provide functional (OCT angiograms) and morphological (OCT B-scans) information and may be performed monthly because it is simple, quick, and noninvasive [15].

A study by de Carlo et al. [9] reported that the sensitivity and specificity of OCTA in detecting CNV secondary to wet AMD were 50% and 91%, respectively. Coscas et al. [16] had compared the OCTA with traditional multimodal imaging in patients with exudative AMD and found that there was high interobserver agreement both for treatment decision in conventional multimodal and for Patterns (I or II) defining on OCTA imaging analysis. However, in our study, the specificity of OCTA for the detection of CNV was 67.6%, with a sensitivity of 86.5%. There are several explanations for this difference, such as the different number of patients, the use of dissimilar inclusion criteria, and the different diagnostic imaging technologies utilized. The six cases (quiescent CNV) that were previously treated with intravitreal anti-VEGF injections classified in our study as false positives had clearly perfused vessels on OCTA, but the FA images showed no leakage. Also, we confirmed that there were no detectable fluid (intra/subretinal/sub-RPE) accumulation on OCT B-scans. We hypothesized that, after treatment, the presence of vessels might indicate persistent CNV on OCTA, which could explain why these patients relapsed and required monthly injections. Upon review of the other five false-positive cases, the OCTA images displayed a homogenous and ill-defined network of vessels, which had a granular appearance at the outer-retina segmentation. As projection artifacts are a limitation of this technology, it is sometimes difficult to distinguish normal physiological vessels from pathological ones. To maximize the sensitivity and specificity of the detection of CNV on OCTA, more advanced software to neutralize projection artifacts while maintaining adequate visibility and intensity of pathological vascular complexes is required. Additionally, readers should be highly trained on how to perform a dynamic evaluation of the OCTA volume, altering the segmentation lines when needed. Of the seven false-negative cases, three of the seven OCTA images had a large amount of subretinal hemorrhage, which limited the penetration of the OCT signal. OCTA may not be ideal for the diagnosis of CNV in these patients. However, it can be used to observe the growth, regression, and recurrence of CNV noninvasively over time after the bleeding has been absorbed. Another limitation of AngioVue OCTA system based on application of an SSADA is that it requires patients to hold their eyes steady for 3-4 seconds and any movement by the patient will cause significant artifact and deterioration of image quality. Patients with very poor acuity cannot maintain fixation for this period and are thus not suitable for this type of examination. The use of eye-tracking software would reduce this artifact.

Jia et al. [17] presented quantitative information on the CNV area and flow in patients with nAMD and reported that higher flow indexes were associated with larger CNV and type II CNV. In our study, we could not detect the density of the blood flow or the flow index due to the limitations of OCT-A technology. However, this did not affect successful visualization of vascular morphology in CNV. OCTA could be used rather than FA as a first step in identifying CNV in patients who are at the transition point from the dry- to wet-type AMD because it is quick and completely noninvasive and can be performed monthly. The sensitivity of OCTA for the detection of CNV in this small case series was 100%. As new-onset CNV secondary to AMD is rarely associated with massive subretinal hemorrhage, it may be particularly suited to diagnosis using OCTA. Future studies with larger sample sizes are needed to confirm the sensitivity rates reported in our study. Figure 4 showed the different morphological patterns probably misdiagnosed in the early detection of nAMD on the OCT. However, OCTA was able to clearly visualize CNV confirmed by FA. Interestingly, in these cases of patients with FA leaky CNV that did not show evidence of fluid on coregistered OCT B-scans, each lesion showed ultrastructural features, such as RPE and/or photoreceptor defects, drusen deposits on the photoreceptor layer and external limiting membrane, or hyperreflective spots, which were previously described in nonneovascular AMD. This suggests that these features may also be of particular value in predicting active CNV lesions that do not show evidence of traditional fluid on OCT B-scans. Similar findings were found by Coscas et al. [16] who had reported that all eyes with sub-, intraretinal, or sub-RPE fluid on OCT B-scan were associated with the active CNV (requiring treatment), but not everyone without fluid accumulation was definitively as quiescent CNV (not requiring treatment).

The finding of common vascular features of prominent vascular loops and anastomotic connections on OCTA among those previously treated with more than 10 intravitreal anti-VEGF injections is intriguing. The trunk vessels were large in diameter, and a variable amount of peripheral anastomosis existed at the outer border of many lesions. Our findings regarding the morphology of the membrane are similar to those described in an earlier study of type 1 neovascularization of AMD [18]. In that study, the authors reported that the large main central vessel trunk could be seen even after the treatment, with the vessels radiating in a branching pattern, in all directions either from the center of the lesion or from one side of the lesion. As is well known, pericytes are an indicator of vessel maturation and have been shown to protect endothelial cells during anti-VEGF therapy. It has been hypothesized that the structure of the central trunk may differ from that of the surrounding finer plexus and may be more resistant to anti-VEGF therapy because the endothelial cells are protected by overlying pericytes [19, 20]. In contrast, tiny branching vessels consist of unprotected endothelial cells, which are more responsive to anti-VEGF therapy. Moreover, increased pressure and flow can cause the branches to expand in size owing to an arteriogenic process [21]. Studying the different morphology of the major central trunk in nAMD complexes may help to explain why some patients react badly to anti-VEGF treatment and others do not.

In our study, the sensitivity and specificity of OCTA in detecting CNV secondary to wet AMD seem to be high, which will likely limit the need for invasive fluorescein and/or indocyanine green angiography. As both functional (blood flow) and morphological (fluid accumulation) information is provided from a single OCT scan, OCT-A may help in the diagnosis of CNV, guiding treatment decisions and monitoring the evolution of CNV. Future studies with larger sample sizes are needed to assess the role of qualitative and quantitative OCTA and improve our understanding of this diagnostic method.

## Figures and Tables

**Table 1:** Detection of eyes with neovascular age-related macular degeneration using optical coherence tomography angiography compared to fluorescein angiography.

	Fluorescein angiography		
	Positive	Negative	Total
Optical coherence tomography angiography			
Positive	45	11	56
Negative	7	23	30
Total	52	34	86

**Table 2:** Sensitivity, specificity, and predictive value of SSADA-OCTA in detecting neovascular age-related macular degeneration.

Imaging modality	Sensitivity (%)	Specificity (%)	Positive predictive value (%)	Negative predictive value (%)
SSADA-OCT	86.5	67.6	80.4	76.7
